# The Biogenesis and Functions of circRNAs and Their Roles in Breast Cancer

**DOI:** 10.3389/fonc.2021.605988

**Published:** 2021-02-25

**Authors:** Liting Tang, Baohong Jiang, Hongbo Zhu, Ting Gao, Yu Zhou, Fuqiang Gong, Rongfang He, Liming Xie, Yuehua Li

**Affiliations:** ^1^ Department of Medical Oncology, The First Affiliated Hospital, University of South China, Hengyang, China; ^2^ Department of Pharmacy, The First Affiliated Hospital, University of South China, Hengyang, China; ^3^ Department of Pathology The First Affiliated Hospital, University of South China, Hengyang, China; ^4^ Key Laboratory of Cancer Cellular and Molecular Pathology in Hunan Province, Cancer Research Institute, Hengyang Medical College, University of South China, Hengyang, China

**Keywords:** circRNA, breast cancer, cell proliferation, apoptosis, drug resistance, prognosis, biomarkers

## Abstract

Recent statistics show that breast cancer is among the most frequent cancers in clinical practice. It is also the second-leading cause of cancer-related deaths among women worldwide. CircRNAs are a new class of endogenous regulatory RNA molecules whose 5’ end and 3’ end are connected together to form a covalently closed single-stranded loop by back-splicing. CircRNAs present the advantages of disease-specific expression and excellent expression stability, and they can modulate gene expression at posttranscriptional and transcriptional levels. CircRNAs are abnormally expressed in multiple cancers, such as breast cancer, and drive the initiation and progression of cancer. In this review, we describe current knowledge about the functions of circRNAs and generalize their roles in various aspects of breast cancer, including cell proliferation, cell cycle, apoptosis, invasion and metastasis, autophagy, angiogenesis, drug resistance, and tumor immunity, and their prognostic and diagnostic value. This may add to a better understanding of the functions and roles of circRNAs in breast cancer, which may become new diagnostic and predictive biomarkers of breast cancer.

## Introduction

Breast cancer is among the most frequent malignant tumors globally and the second top cause of cancer fatalities among women globally (1, 2). It poses a serious threat to lives of women. Cases of breast cancer have been mounting over the past decade (3, 4). Breast cancer is a complex and highly diverse disease (5, 6). According to its histological features, breast cancer can be grouped into four distinct molecular sub-kinds, i.e., luminal A, luminal B, HER-2-overexpressing, and triple-negative breast cancer (TNBC), based on the expression of ER, PR, HER-2, and Ki-67 (5). TNBC is responsible for 15%–20% of all breast cancers and remains the most challenging subtype of breast cancer. For a specific molecular target therapy in the prognosis of patients with TNBC, there is seldom clinically meaningful improvement; therefore, TNBC is one of the worst prognosis among breast cancer subtypes of all types due to the high propensity for progression, difficulty of identifying effective targets, and lack of specific targeted therapy, leading to a greater recurrence and metastasis potential and shorter overall survival (7, 8). The heterogeneity of breast cancer causes great confusion in diagnosis and treatment and affects the survival rate, treatment response, and tumor growth rate (9, 10). Some scholars in the United States have found that if early detection of breast cancer patients and high-quality treatment services can be provided, this can accelerate the reduction of breast cancer mortality (11). Therefore, the betimes diagnosis of breast cancer is a pivotal component of breast cancer treatment. However, because early breast cancer usually does not present the typical symptoms and signs of breast cancer and women often ignore breast self-examination and clinical examination, breast cancer is still generally diagnosed at a late stage (12, 13). The complexity of the pathogenesis of breast cancer and the diversity of its molecular characteristics and clinical manifestations lead to inconsistent prognosis in the majority of patients, resulting in an inability to achieve the desired effect. Therefore, it is of great significance to study the molecular mechanism and signal transduction pathway of breast cancer progression and to identify early diagnostic biosignatures and novel treatment targets for the more effective treatment of breast cancer.

CircRNAs constitute a new class of endogenous regulatory RNAs exhibiting covalently closed continuous ring structures, no 5’ to 3’ polarity and polyadenylate tails. Because circRNAs lack free ends, they are not easily broken down by nucleic acid exonucleases and are more stable relative to linear RNAs. CircRNAs not only present the advantage of stability but also show the unique advantages of a high richness and breadth (14–17). Numerous circRNAs are expressed in various tissues of mammals, and 30,000 different circRNAs have been found in human tissues alone (18). CircRNA research has led to many surprising findings, and circRNAs play very important roles in biology and pathobiology (19). Recently, a mounting number of circRNAs have been discovered and reported to exhibit different expression levels in breast cancer. These abnormal circRNAs are involved in the biological processes of breast cancer consisting of cell proliferation, cell death, metastasis, drug resistance, as well as its prognosis (6, 20). Moreover, because of their high degree of conservation and tissue specificity, circRNAs have the capacity to serve as a tumor marker, as well as therapeutic target for breast cancer. Moreover, the regulation of circRNA may be an underlying mechanism of breast cancer and an indispensable component of its diagnosis and treatment.

## Biogenesis of circRNA

**Figure 1:**
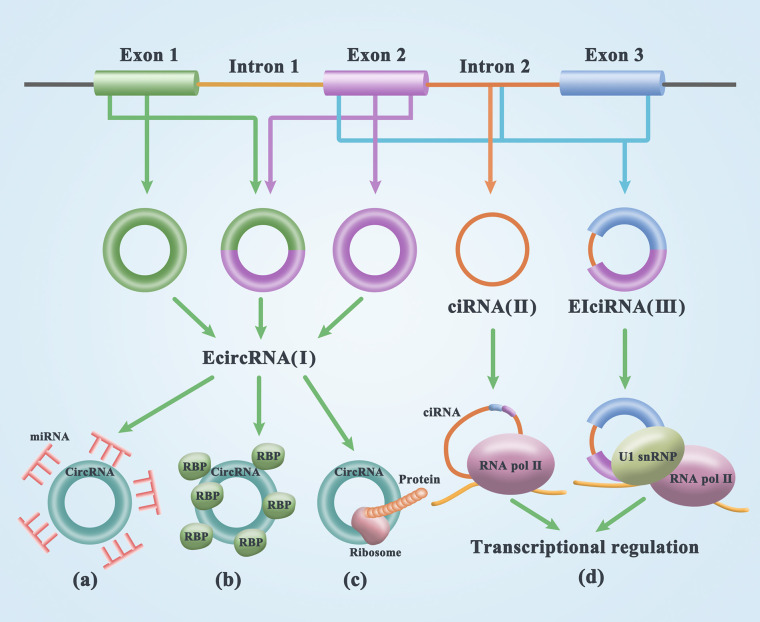
Biogenesis and functions of circRNAs. (I) exon circRNA (EcircRNA) is formed by lariat-driven circularization and intron pairing-driven circularization. (II) circRNA (ciRNA) is the source of introns generated by the partial degradation of introns after the formation of the lasso structure. (III) circRNA (EIciRNA), which is composed of exons and introns, is cyclized during splicing. **(A)** The adsorption of miRNA by circRNA results in the formation of the circRNA/miRNA/mRNA regulatory axis by reducing miRNA expression and releasing mRNA from functional inhibition. **(B)** CircRNA can act as a RNA-binding protein sponge, binding with RBPs to form an RNA-protein complex (RPC). RBPs are mainly involved in posttranscriptional regulatory processes. **(C)** A small number of small open reading frames in circRNAs actually have the potential to encode peptides or proteins. **(D)** CircRNAs in the nucleus is involved in transcription regulation.

CircRNA was first discovered in plant viruses by Sanger’s group in 1976. Studies have shown that circRNAs are circular, single-stranded, and covalently closed RNA biomolecules (21). Initially, most circRNA molecules were thought to be byproducts of transcriptional noise or the cell splicing machinery (22–24). Through mutation analysis of circRNA expression vectors and the treatment of HeLa cells with isoginkgetin, a splicing inhibitor that blocks spliceosome assembly, it was found that the biogenesis of circRNA is dependent on the typical splicing mechanism (19) resulting from the reverse splicing of the pre-mRNA (25). CircRNAs are widely distributed and are a diverse family of natural components of endogenous noncoding RNAs that are involved in miRNA inhibition, epithelial-mesenchymal transformation, and tumorigenesis, potentially regulating mammalian gene expression (26, 27). The composition of circRNAs can be divided into three categories. (1) Exonic circRNAs (EcircRNAs) are mainly generated in two ways: lariat-driven circularization relied on exon skipping and intron pairing-driven circularization. The former is formed by excision of introns after covalent binding of exons and splicing recipients, while the latter is formulated by excision of introns after two introns are complementary paired to form a ring structure (28) [**Figure 1**(I)]. (2) circRNA (ciRNA) is the source of introns generated by the partial degradation of introns after the formation of the lasso structure [**Figure 1**(II)]. (3) circRNA (EIciRNA), which is composed of exons and introns, is cyclized during splicing. In 2013, Jeck proposed that exon skipping and intron pairing reduce the distance between splicing sites and promote the reverse splicing of premRNA. This leads to the deletion of the 3’ and 5’ ends of circRNAs (29) [**Figure 1**(III)]. In general, linear RNA production involves the removal of introns and the sequential binding of exons. Thus, circRNA formation is precisely controlled. Two introns located on the side of the ring exon can increase Alu duplication, thus improving the efficiency of ring formation (25).

Compared with linear homologous RNA, circRNAs exhibit different reverse splicing and alternative RNA splicing patterns. The selection of the selective backshear site is related to the competition of presumed RNA pairs between introns containing selective backshear sites. The diversity of selective reverse splicing and alternative RNA splicing in circRNAs provides a valuable resource for describing the complexity of circRNA formation (30, 31). The majority of circRNAs in the cytoplasm come from exon sources. Intron sources and circRNAs consisting of exons, as well as introns are mainly located in the nucleus. It has been proven that circRNA is widely expressed in a variety of organisms in a manner that is specific to species, tissues, diseases, and developmental stages and is an important biological regulatory factor that serves a pivotal role in the pathogenesis and progression of breast cancer (32). CircRNA has been shown to regulate all attributes of breast cancer development (such as cell proliferation, apoptosis, metastasis, drug resistance, and prognosis). CircRNAs are highly stable and can be secreted outside of the body, with no cells being found in the saliva and blood plasma (25). Further investigation of circular RNA interactions with tumors to understand their crosstalk and regulatory modes may identify promising therapeutic targets and biomarkers of disease (33) for evaluation and prognosis prediction in the early diagnosis and treatment of tumors and tumor gene therapy (34, 35).

## The Functions of circRNAs

### miRNA Sponging

MicroRNA (miRNA) is an important posttranscriptional regulator of gene expression acting on target sites in untranslated regions of mRNAs through direct base pairing. In 2011, Salmena et al. (36), for the first time, advanced the theory of ceRNA. They suggested that one miRNA can modulate diverse target genes and that the same target gene can be modulated by different miRNAs. These RNAs regulated by the same miRNA exhibit a competitive relationship and are therefore referred to as ceRNAs. Some circRNAs possess miRNA-binding sites and serve as ceRNAs to regulate miRNA-inhibitory gene expression, thereby upregulating target gene expression. As a widely studied type of competitive endogenous RNA, circRNA can regulate multiple biological processes and play an important role in regulating human gene expression, cancer occurrence, and progression (37, 38). Since miRNAs are key regulators, their deregulation can lead to gene deregulation, which can lead to serious diseases such as cancer. CircRNA can act as a miRNA sponge, which is the most studied function of circRNA. The strongest evidence of this sponging effect comes from the first discovery of the binding of CDR1as to an miRNA that contains sequences matching 74 miR-7 seeds and is tightly bound by the AGO protein (miRNA-binding protein), which strongly inhibits the activity of miR-7 in nerve tissue and leads to an increase in the miR-7 targeting level, thus affecting the development of tumors (31, 39). CircRNAs regulate miRNA expression in the cytoplasm by acting as miRNA sponges and absorbing miRNAs or other targets to regulate tumor metabolism (7, 40, 41). The adsorption of miRNA by circRNA results in the formation of the circRNA/miRNA/mRNA regulatory axis by reducing miRNA expression and releasing mRNA from functional inhibition (42) (**Figure 1A**). The circRNA/miRNA/mRNA axis has been shown to be associated with the development of 427 different diseases (43). Most circRNAs accumulate only a few molecules per cell. To act as miRNA sponges, these circRNAs may require additional miRNA-binding sites at very low levels (44). Hansen et al. (45) have demonstrated that ciRS-7 is a distinct sponge for miR-7, repressing the expression of diverse oncogenes modulated by miR-7, indicating that the ciRS-7/miR-7 cascade serves a pivotal role in cancer-linked cascades, as well as clinical applications.

### Combination With RBPs

CircRNA can act as a RNA-binding protein sponge, binding with RBPs to form an RNA-protein complex (RPC), which serves a crucial role in a multiple cellular processes. RBPs are mainly involved in posttranscriptional regulatory processes, such as the selective splicing, transcription, and translation of RNA, thus affecting the transcription of linear parental genes, regulating the expression of related proteins, and playing a vital role in the progression of tumors (46, 47) (**Figure 1B**). Many studies have confirmed that many circRNAs interact with RBPs. For example, circRNA CDR1as is widely related to the AGO protein. CircRNA Mbl contains conserved muscleblind (MBL) protein-binding sites. In addition, circRNA PABPN1 can bind to HuR, prevent HuR from binding to PABPN1 mRNA and reduce the translation of PABPN1 (48).

### Translation of Proteins or Peptides

Conventional wisdom is that circRNAs are nonprotein-coding RNAs, but recent evidence suggests that a small number of small open reading frames in circRNAs actually have the potential to encode peptides or proteins. These peptides/proteins serve a pivotal role in modulating the energy biometabolism of tumors, the transformation of cancer cells from the epithelium to the stroma, the stableness of the c-myc oncoprotein, as well as the ubiquitination and degeneration of proliferating cell nuclear antigen (PCNA). These peptides/proteins further constitute prospective drug targets for repressing tumor multiplication or a biomarker for estimating the prognosis of cancer patients (49, 50) (**Figure 1C**). Researchers have found that circRNADb contains 32,914 human circRNAs, among which 16,328 may contain ORFs with at least 100 amino acids (51). Zhou et al. (43) found that circ-ZNF609 could be translated into proteins during the *in vitro* differentiation of mouse and human myoblasts by shear-dependent and cap-independent methods.

### Transcription Regulation

Research evidence chronicles that circRNAs participate in the modulation of transcription, or alternative RNA splicing (**Figure 1D**). For instance, Ashwal-Fluss et al. detailed that circMBL is produced by the 2^nd^ exon of MBL, which competed with the canonical pre-mRNA splicing. The flanking intron of circMBL and circMBL has conservative MBL binding sites, which are firmly and distinctly bound by MBL. The regulation of MBL level remarkably influences the generation of circMBL, which relies on the binding site of MBL in the lateral intron sequence. These data imply that general splicing parameters, like MBL, may affect alternative RNA splicing, regulate circRNA biogenesis, and canonical splicing (52). Eukaryotic transcription is mainly modulated at the activation stage, forming a pre-activation complex at the enhancer. All known transcription factors aggregate to further stabilize the complex and stimulate the transcription rate. Nuclear run-on tests were conducted with nuclei isolated from circEIF3J, as well as circPAIP2 silenced cells. Consequently, it was chronicled that the silencing of circEIF3J, as well as circPAIP2 resulted in down modulated EIF3J and PAIP2 transcription. Nonetheless, the silencing of EIF3J, as well as PAIP2 using siRNA exhibited no remarkable influence on their transcription. Both circEIF3J and circPAIP2 were reported to be co-localized with the genomic loci of their parental genes as revealed by RNA-DNA double FISH. These data collectively indicates that circEIF3J and circPAIP2 may regulate the expression of their aboriginal genes in cis (53). Exon-intron circRNAs (EIciRNAs) in humans facilitate circRNA host gene transcription *via* correlation with U1 snRNP. Similarly, EIciRNAs have been reported in plants and participate in gene modulation in biotic stress reactions (54). CircSEP3 in Arabidopsis stems from the SEPALLATA3 (SEP3) gene modulates transcription, as well as splicing of their linear mates [**Figure 1D)**]. CircSEP3 binds firmly to its correlated DNA locus to generate an RNA: DNA hybrid; however, the counterpart linear RNA binds much more weakly to the DNA. The formation of circRNA: DNA hybrid leads to transcriptional pausing and results in the generation of differentially spliced SEP3 mRNA with exon skipping (55). Collectively, these data imply that circRNAs can coordinate gene expression at both splicing, as well as transcription levels. In maize, three kinds of circRNAs are generated from CRM1, each with varying sizes however the same back-splicing region. These circRNAs have the potential to interact and bind to centromeric chromatin *via* the R-loops. The two R-loop regions in a single circRNA enhance the generation of chromatin loops. RNAi targeting the back-splicing region of the circNAs, diminishes the contents of R-loops, as well as the chromatin loops generated by the circRNAs; nonetheless, the Rloops of the two forms of linear RNAs with similar R-loop generation regions escalated. The elevated contents of R-loops and diminished contents of chromatin loops in the CRM1 sites result in a diminished CENH3 localization in the RNAi plants. Besides, the back splicing process in retrotransposons was reported to be conserved in various species of crops (56).

**Figure 2:**
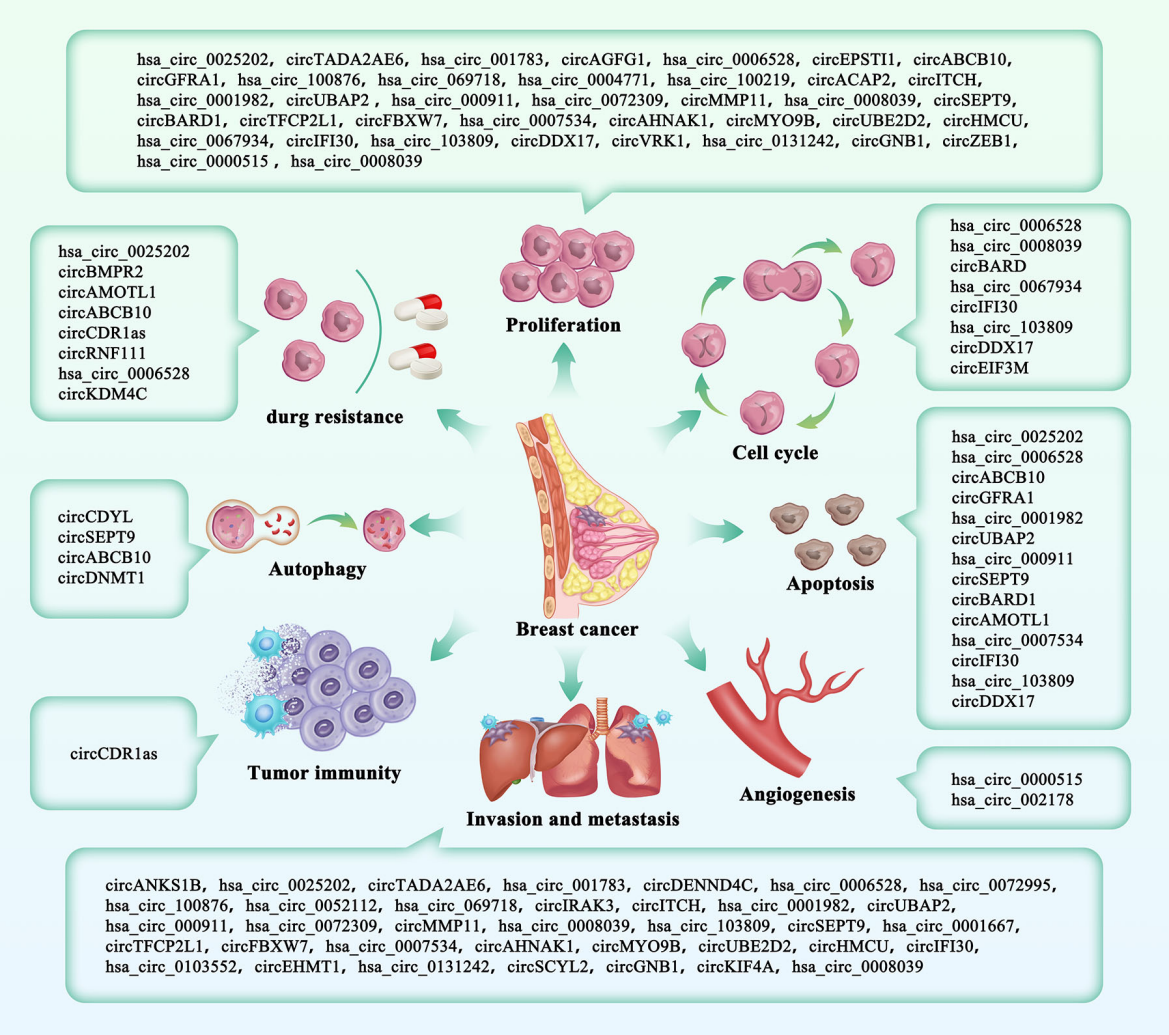
The roles of circRNAs in breast cancer. CircRNAs included in our study were divided into eight parts according to their clinical characteristics.

## CircRNA and Breast Cancer

### Proliferation

In eukaryotes, a decrease in CDK1/cyclinB1 complex formation is associated with G2/M accumulation. P21 is a mitotic regulator that can block the activation of CDK1 by binding with the CDK1/cyclinB1 complex and inhibiting the growth and proliferation of breast cancer cells in G2/M phase (57). CircDDX17 inhibits cell proliferation by regulating cell cycle-related factors (CDK1 and P21) (58). circRNA_ 103809 overexpression can directly regulate the function of miR-532-3p and block G2/M phase, and it can repress the proliferation and metastasis of BC cells by interfering with the EMT signaling cascade (59). Zheng et al. (60) designed a circSEPT9 overexpression vector and constructed three siRNAs targeting the circSEPT9 junction regions. The qRT-PCR findings revealed that the circSEPT9 expression was markedly up-modulated or down-modulated in the cellular TNBCs transfected with the designated vectors or siRNA segments, respectively. The colony generation, CCK-8, as well as EdU tests, was utilized to assay for cell viability. The findings revealed circSEPT9 silencing markedly repressed TNBC cell proliferation. *In vitro* findings indicated that circHMCU remarkably enhances breast tumor cell proliferation by influencing the G1 stage cell cycle checkpoint. Further *in vivo* reports have detailed that the excessive expression of circHMCU contributes to aggressive proliferation (61). Based on the *in vitro* studies, circ-VRK1 expression is down modulated in BC cell lines comprising MDA-MB-231, BT474 and MDA-MB-453 relative to the normal immortalized breast epithelial cell lines (MCF10A), and circ-VRK1 represses tumor cell multiplication (62). Studies have chronicled that hsa_circ_0131242 silencing represses breast cancer cell growth. Bioinformatics estimations and luciferase enzyme reporter assessments posited that hsa_circ_0131242 sponges hsa-miR-2682. Besides, the co-transfection of hsa-miR-2682 repressor and si-hsa _circ_0131242 abolishes cell proliferation, as well as migration in the BT549 and MDA-MB-468 cell lines (63).

### Cell Cycle

Many studies have shown that the cell cycle serves as the convergence point of oncogenic signaling cascades, and atypical cell cycle development is an important characteristic of cancer. Tumor cells, whose cell cycle is usually regulated in a disordered state, can easily avoid the restriction point without any preparation, resulting in unrestricted proliferation (64). Mechanistic assessment suggests that circAGFG1 may serve as a competitive endogenous RNA (ceRNA) for miR-195-5p, mitigating the inhibitory influence of miR-195-5p on its target cyclin E1 (CCNE1). CircAGFG1 silencing may repress the segregation of E2F1 with RB through CCNE1-triggered changes in RB phosphorylation, resulting in decreased transcriptional activity of E2F1, as well as G1/S cell cycle arrest. That is, circAGFG1 is a ceRNA that promotes CCNE1-mediated proliferation in TNBC by inducing miR-195-5p (65). The depletion of hsa_circ_0008039 distinctly represses the proliferation (*in vivo*), inhibits cell cycle progression, and reduces migration of BC cells by acting as ceRNA to sponge miR-432-5p and downregulated E2F3 (3). After circ_0067934 were knocked out in MCF-7 and SKBR3 cells, the proportion of G0/G1 cells escalated and the proportion of S cells diminished. Studies showed that BC cell cycle was regulated after the downregulation of circ_0067934 *in vitro*. Further experimental results showed that circ_0067934 could regulate cell cycle by up-regulating Mcl-1 (66). CircEIF3M was highly up-modulated in the TNBC cells, as well as tissues and remarkably enhanced the development of TNBC cells. Additionally, circEIF3M, as the ceRNA of miR-33a, activates the expression of CCND1, which synergistically regulates cell cycle progression with cyclin-dependent kinase 4 (CDK4). The activity of CCND1-CDK4 is dysregulated in a variety of cancers, such as melanoma and breast cancer, and circEIF3M downregulation can result in cell cycle arrest (67).

### Apoptosis

Apoptosis is a type of programmed active death of cells that occurs under gene regulation, usually characterized by nuclear enrichment, wrinkling, membrane foaming, and DNA fragmentation. Under physiological conditions, apoptosis is beneficial to maintain the stability of the intracellular environment and allow better adaptation to changes in the living environment (68). The occurrence of tumors is mainly due to uncontrolled proliferation and apoptosis inhibition, so inducing tumor cell apoptosis is an important strategy to prevent the occurrence and development of tumors. Chen et al. (69) studied the expression profile of human circRNA in TNBC tissues and uncovered circEPSTI1 (hsa_ circRNA_000479) as a remarkably up modulated circRNA. By knocking out circEPSTI1 in three TNBC cell lines and conducting MRE examination and luciferase enzyme reporter gene analysis, circEPSTI1 was found to bind to miRNA as a common target gene of miRNA sponges and miRNAs. CircEPSTI1 contents in 240 TNBC patients were assessed by ISH, and the findings showed that the downregulation of the circEPSTI1 gene induced apoptosis. Hsa_circ_0007534 expression was markedly increased in BC tissues and cell lines, while miR-593 expression was remarkably decreased. Down-regulation of hsa_circ_0007534 can promote BC cell apoptosis. Hsa_circ_0007534 was established as the target of miR-593, and the expression of miR-593 in BC cells could be inversely modulated by hsa_circ_0007534 (70).

### Invasion and Metastasis

Breast cancer metastasis is among the leading causes of fatalities in cancer patients. It has been reported that the 5-year survival rate of patients showing metastasis at the time of diagnosis is only 26% (71). Epithelial to mesenchymal transformation (EMT) is a key step in breast cancer cell metastasis, in which epithelial cells progressively lose their polarity and adhesion ability, while mesenchymal characteristics are acquired through the downregulation of epithelial markers such as E-cadherin and upregulation of mesenchymal biomarkers such as N-cadherin as well as vimentin. At the same time, it has been extensively accepted that TGF-signaling is the main EMT inducer by activating the initiation of the Smad complex, which translocases into the nucleus to modulate gene expression. EMT is an important mechanism of malignant tumor metastasis, and this signaling pathway may be regulated by circRNA (9). CircANKS1B makes full use of miR-148a-3p, as well as miR-152-3p to escalate the expression of the transcription factor USF1; therefore, upregulating the expression of TGF-β1 and activating the TGF-β1/Smad signal to promote EMT (72). Functional studies showed that the downregulation of hsa_circ_001569 significantly inhibited the growth and metastasis potential of BC cells. Studies of molecular mechanisms showed that the inhibition of hsa_circ_001569 inhibited the activation of the PI3K/AKT signal in BC cells. Hsa_circ_001569 may promote the development of BC by regulating the PI3K/AKT pathway (73). Some researchers have used MCF-7, MDA-MB-231, qRT-PCR, as well as western blotting to analyze the expression profile of circRNA chips and quantitatively express circASS1 and its parent gene ASS1. Wound healing, migration, and infiltration tests were conducted. A luciferase detection system was employed to assess the hidden miRNAs. Results revealed that CircASS1 expression in MDA-MB-231 cells was down modulated relative to the MCF-7 cells, and the overexpression of CircASS1 repressed the infiltration and migration. CircASS1 repressed the infiltration and migration of breast cancer cells (74). RT-PCR was used to quantitatively detect the expression of circRNA_100876 in 50 pairs of BC tissue samples and corresponding adjacent samples. Additionally, qRT-PCR was further employed to detect the expression level of circRNA_100876 in BC cell lines. In addition, the circRNA_100876 knockout model was constructed in a lentivirus and transfected into BC cells. Subsequently, the effects of circRNA_100876 on BC cell function were analyzed using cell count kit-8, Transwell, and clone formation experiments. The interaction between circRNA_100876 and microRNA-361-3-p was verified by a luciferase enzyme reporter assay and cell reverse assay. The qRT-PCR results showed that the expression of circRNA_100876 in BC tissues was remarkably higher than that in paracancer tissues and that its expression in patients with distant metastasis was distinctly elevated relative to the patients without distant metastasis (75). MMPs, especially MMP9 and MMP2, are important biomarkers for breast cancer migration and invasion. By measuring MMP levels and conducting transwell measurements, we found that increased MMP2 and MMP9 levels in cells under hypoxia increased cell migration and invasion, while the silencing of circDENND4C inhibited cell migration. We hypothesized that the antimetastatic effect of circDENND4C silencing may be related to epithelial mesenchymal transformation, which requires further study in the future. These findings imply that circDENND4C is a therapeutic target in breast cancer patients (76). Circ_0006528 can suck the endogenous miR-7-5p and repress its bioactivity. We also identified Raf1, which activates the MAPK/ERK signaling cascade, as the target of miR-7-5p, and established that circ_0006528 enhances the growth, infiltration, and migration of breast cancer and activates the MAPK/ERK cascade by enhancing the expression of Raf1 (77).

### Autophagy

Autophagy is a conserved, ubiquitous process and participates in an energy cycle that can deliver damaged organelles, misfolded proteins and intracellular components to lysosomes for degradation (78, 79). Autophagy leads to the dysregulation of various pathological behaviors in eukaryotic cells, promoting disease progression, including that of cancer. Autophagy provides energy and helps keep cancer cells alive when they are under stress (lack of oxygen and hunger) (80). mTOR is a downstream molecule of AKT1, and the AKT/mTOR pathway is one of the classical signaling pathways mediating tumor metabolic homeostasis. The AKT/mTOR axis can promote anabolism, including protein synthesis, and block catabolic activity, such as autophagy, which is ultimately beneficial to the growth of GC cells. mTORC1 slows the aging of GC cells by inhibiting catabolic activity (including autophagy), thus promoting cell growth. mTORC1 phosphorylates ULK1 to prevent its activation by AMPK, a key activator of autophagy (81). The effects of circSEPT9 knockdown on TNBC autophagy have been described in the relevant literature. Immunofluorescence analysis showed that circSEPT9 depletion induced the generation of LC3-II puncta and autophagy accumulation. The total number of LC3-II puncta per cell in the si-circSEPT9 group was remarkably greater relative to the control group. After the knockout of circSEPT9, the transformation of the autophagy biosignature LC3 from LC3-I to LC3-II was markedly elevated, and the levels of the autophagy-correlated proteins ATG5 and ATG7 were distinctly increased (60). Circ-ABCB10 has been found to mediate the autophagy of BC cells through the Let-7A-5P/DUSP7 axis (82). We additionally established that ectopically expressed circ-DNMT1 could interact with p53, as well as AUF1, enhancing the nuclear translocation of both proteins. Nuclear translocation of p53 triggered cellular autophagy while AUF1 nuclear translocation diminished DNMT1 mRNA instability, causing elevated DNMT1 translation. From here, functional DNMT1 could then translocate into the nucleus, repressing p53 transcription. Highly expressed circ-DNMT1 binds and regulates oncogenic proteins in breast cancer cells. Ectopic circ-DNMT1 increases the proliferation and survival of breast cancer cells by triggering autophagy (83).

### Angiogenesis

Angiogenesis serves a pivotal role in tumor growth, as well as metastasis. It ensures a continuous blood supply for the abnormal proliferation and expansion of tumor cells and promotes tumor cell growth (84–86). The signaling pathways contributing to tumor angiogenesis include those involving the hypoxia-inducible factor (HIF) family, platelet-derived growth factor (PDGF), vascular endothelial growth factor (VEGF) family, TGF-β, tumor necrosis factor-α (TNF-α), interleukin, fibroblast growth factor (FGF), αvβ3 integrins, Eph-B4/ephrinB2, VE-cadherin, plasminogen activator/plasmin, matrix metalloproteinases (MMPs), and three UTRs of CYP4Z2P and CYP4Z1-3′UTR pseudogenes, which promote breast tumor angiogenesis by isolating miRNAs, including miR-211, miR-125a-3p, miR-197, miR-1226, as well as miR-204. These ceRNAs therefore allow CYP4Z1 translation and jointly promote breast cancer angiogenesis by activating the PI3K and ERK1/2 cascades (87). Liu et al. (88) confirmed through experiments that the downregulation of hsa_circRNA_002178 can inhibit aggressive malignant behaviors such as angiogenesis. At the same time, some scholars found that hsa_circ_0000515 and miR-296-5p modulated BC cell angiogenesis stimulated by CXCL10 specifically (89).

### Drug Resistance

Several studies have analyzed the expression of circRNA in resistant breast cancer and found that circRNA may serve a role in enhancing or reversing chemical drugs resistance in breast cancer. At present, chemotherapy and endocrine drugs play a vital role in the therapy of breast cancer after surgery, but there are still some patients showing drug resistance, which hinders their treatment. TNBC is the most lethal sub-kind of breast cancer, characterized by high diversity and invasiveness and a lack of therapeutic options. Chemotherapy remains the standard regimen for TNBC therapy, but patients unfortunately often develop drug resistance (90). Drug resistance constitutes the main factor that limits systemic chemotherapy for advanced metastatic breast cancer and is one of the main reasons for the low relapse-free survival rate in breast cancer and other cancers. Multiple genes serve an important role in the occurrence and progression of drug resistance, such as MDR1, which encodes P-glycoprotein, and multidrug resistance-related proteins (MRPS). Breast cancer drug-resistant protein (BCRP) belongs to the ATP binding box (ABC)-superfamily multidrug exudation pump, and glutathione s-transferase (GST) and O^6^-methylguanine DNA methyltransferase (MGMT) are a phase II detoxification enzyme and DNA repair enzyme, respectively. The intracellular accumulation of various chemotherapy drugs is reduced (87, 91) and circ_MTO1 (hsa_circ_0007874) is downregulated in monastrol drug-resistant cell lines (MDA-MB-231R and MCF-7R), which regulates the TRAF4/Eg5 axis by targeting the Eg5 protein, binding TRAF4 to the Eg5 gene and thereby inhibiting BC cell activity and promoting monastrol-induced cytotoxicity (92). By upregulating miR-7, the repression of circRNA CDR1as can escalate the chemical sensitivity of 5-FU-resistant BC cells, implying that CDR1as may be a prospective tool for establishing and optimizing chemotherapy approaches for BC (93). To investigate the role of circRNAs in tamoxifen resistance, we established a tamoxifen-resistant MCF7/TR cell line and screened its circRNA expression profile by RNA sequencing. Hsa_circ_0025202 is a significantly reduced circRNA. Functional studies have shown that tumor repression and the tamoxifen sensitization of are achieved through the hsa_circ_0025202/miR-182-5p/FOXO3a axis. Hsa_circ_0025202 has an anticancer effect in HR-positive breast cancer and can be used as a new biomarker for the treatment of tamoxifen breast cancer (94). Gao et al. (95) investigated the expression of circ 0006528 in ADM-resistant cell lines (MCF-7/ADM and MDA-MB-231/ADM) and tissues (n = 40) using qRT-PCR. The findings showed that the circ 0006528 expression in the ADM-resistant cell lines and tissues were elevated relative to the respective ADM-sensitive groups. This suggests circRNAs may participate in breast cancer chemoresistance. It has also been found that circKDM4C is down-regulated in adriamycin resistant breast cancer cells. CircKDM4C silencing or overexpression led to significant increase or decrease of adriamycin resistance (96).

### Tumor Immunity

The immune response associated with cancer can protect the host by destroying or inhibiting the growth of cancer cells. Additionally, the development of a tumor-specific adaptive immune response can be promoted by selecting tumor escape variants or establishing certain conditions in the tumor microenvironment (97). CircRNA is involved in mediating the immune response. Studies have shown that circRNA CDR1as may serve a distinct role in immune and stromal cells invasion in tumor tissue of BC, particularly those of CD8+ T cells, activated NK cells, M2 macrophages, cancer-correlated fibroblasts (CAFs), and endothelial cells (98).

## CircRNAs Act as Diagnostic and Prognostic Biomarkers

Prognostic assessment plays an important role in prolonging the survival of cancer patients. Many studies have shown that circRNAs are involved in various pathological processes of breast cancer. Therefore, circRNAs are attracting increasing attention as potential prognostic biomarkers of breast cancer (23). Yang et al. (99) knocked out circ_0103552 in MCF7 cells and evaluated the effect of circ_0103552 on cell viability by using cell count kit 8 (CCK8) and clone formation experiments. The results showed that the cell viability of the MCF7 cell line decreased significantly after circ_0103552 was silenced. In addition, the increase of circ_0103552 was closely related to clinical severity in BC patients (including advanced TNM and positive lymph node infiltration). The upregulation of circ_0103552 is an independent prognostic indicator for patients undergoing therapeutic surgery. KIF4A, a member of the drive protein family, has been identified as an oncogene that is overexpressed in many malignancies, including breast cancer. High KIF4A expression is significantly associated with poor prognosis in a variety of cancers. KIF4A is critical to cancer progression and shows potential as a prognostic biomarker and therapeutic target. CircKIF4A regulates the expression of KIF4A by sponging miR-375, thus exerting its regulatory function in TNBC. The circKIF4A-miR-375-KIF4A axis regulates the TNBC process through a ceRNA-related mechanism. Therefore, circKIF4A can be used as a prognostic biomarker and therapeutic target for TNBC (100). CircHMCU is a miRNA sponge of the let-7 family, which plays a carcinogenic role in breast cancer and can be used as a new biomarker for the diagnosis and prognosis of BC (61). Some studies have demonstrated that hsa_circ_0104824 is down-regulated in BC tissues and peripheral blood of patients. ROC curve analysis shows that hsa_circ_0104824 has high diagnostic value for BC. Therefore, it can be speculated that hsa_circ_0104824 can be used as a potential biomarker for the diagnosis and treatment of BC (101). Jia et al. (102) have demonstrated that hsa_circ_0007255 plays a new oncogene role in the occurrence and development of BC by regulating the miR-335-5p/SIX2 axis and is expected to become a biomarker for the treatment of BC.

## Conclusions

BC is an important disease that endangers women’s health, so it is very important to study BC in depth. CircRNA has attracted the attention of researchers due to its unique properties and has become a new research hotbed in recent years. Mounting number of studies have shown that circRNA can be specifically expressed in tissues and differentially expressed in tumor and nontumor tissues. Because of the large number of circRNAs in saliva, blood, and exosomes, circRNAs can be employed as potential biosignatures for the diagnosis or prediction of disease, especially related to the occurrence, development, and prognosis of cancer. Some circRNAs show stable abnormal expression in BC that can be detected with good sensitivity and specificity and have been proven to be linked to a dismal prognosis, providing a new option for the early diagnosis of BC and the design of prognostic markers. However, there are still many problems surrounding circRNAs that require further clarification: 1. Although circRNAs play multiple roles, little is known about their roles in BC. 2. The mechanism of action of circRNAs still need to continue to be explored, and their clinical application will require the continuous efforts of more researchers and clinical workers. 3. In studies on the mechanism of the circRNA regulation of BC progression, single tumor cells have been targeted, but it is unclear whether circRNA is abnormally expressed in the tumor microenvironment, whether it is abnormally expressed in different cells in the microenvironment, and whether these circRNAs can be transmitted to different cells to promote tumor progression. 4. Despite advancements in diagnosis and therapy, the treatment of locally advanced breast cancer (LABC) remains a major clinical problem.

## Author Contributions

LT, BJ, LX, and YL conceived this manuscript. LT, BJ, and HZ collected data and drafted the manuscript. HZ, YZ, and FG drew the figures. LX and YL contributed to the revision of figures. RH, YL, and LX contributed to the critical review of the manuscript and revised the final version of the manuscript. All authors contributed to the article and approved the submitted version.

## Funding

This work was supported by fund from the National Natural Science Foundation of China (NO.81902707), the Hunan Provincial Natural Science Foundation (NO.2018JJ6092 and 2019JJ50537), the Health and Family Planning Commission of Hunan Province (NO. B2019120, B2019124, and C2019114), and the Science and Technology Project of Hengyang (NO. S2018F9031021272).

## Conflict of Interest

The authors declare that the research was conducted in the absence of any commercial or financial relationships that could be construed as a potential conflict of interest.
